# Genetic diversity, connectivity and gene flow along the distribution of the emblematic Atlanto-Mediterranean sponge *Petrosia ficiformis* (Haplosclerida, Demospongiae)

**DOI:** 10.1186/s12862-018-1343-6

**Published:** 2019-01-16

**Authors:** Ana Riesgo, Sergi Taboada, Rocío Pérez-Portela, Paolo Melis, Joana R. Xavier, Gema Blasco, Susanna López-Legentil

**Affiliations:** 1 Department of Life Sciences, The Natural History Museum, Cromwell Road, London, SW7 5BD UK; 2 Department of Biology (Zoology), Autonomous University of Madrid, Faculty of Sciences, Cantoblanco, 28049 Madrid, Spain; 3 Department of Geology and Biology, Physics and Inorganic Chemistry, King Juan Carlos I University, C/ Tulipán s.n, 28933 Móstoles, Madrid Spain; 4 Department of Evolutionary Biology, Ecology and Environmental Sciences, Faculty of Biology, University of Barcelona, Avda. Diagonal 643, 08028 Barcelona, Spain; 5 CIIMAR, Interdisciplinary Centre of Marine and Environmental Research of the University of Porto, 4450-208 Matosinhos, Portugal; 6 Department of Biology, KG Jebsen Centre for Deep-Sea Research, University of Bergen, Thormøhlensgate 53A, 5006 Bergen, Norway; 7 Department of Biology and Marine Biology, University of North Carolina Wilmington, 5600 Marvin K. Moss Lane, Wilmington, NC 28409 USA

**Keywords:** Population genetics, Migration, Porifera, Dispersal, Inbreeding, Bottleneck, Genetic differentiation, Hardy-Weinberg equilibrium

## Abstract

**Background:**

Knowledge about the distribution of the genetic variation of marine species is fundamental to address species conservation and management strategies, especially in scenarios with mass mortalities. In the Mediterranean Sea, *Petrosia ficiformis* is one of the species most affected by temperature-related diseases. Our study aimed to assess its genetic structure, connectivity, and bottleneck signatures to understand its evolutionary history and to provide information to help design conservation strategies of sessile marine invertebrates.

**Results:**

We genotyped 280 individuals from 19 locations across the entire distribution range of *P. ficiformis* in the Atlanto-Mediterranean region at 10 microsatellite loci. High levels of inbreeding were detected in most locations (especially in the Macaronesia and the Western Mediterranean) and bottleneck signatures were only detected in Mediterranean populations, although not coinciding entirely with those with reported die-offs. We detected strong significant population differentiation, with the Atlantic populations being the most genetically isolated, and show that six clusters explained the genetic structure along the distribution range of this sponge. Although we detected a pattern of isolation by distance in *P. ficiformis* when all locations were analyzed together, stratified Mantel tests revealed that other factors could be playing a more prominent role than isolation by distance. Indeed, we detected a strong effect of oceanographic barriers impeding the gene flow among certain areas, the strongest one being the Almeria-Oran front, hampering gene flow between the Atlantic Ocean and the Mediterranean Sea. Finally, migration and genetic diversity distribution analyses suggest a Mediterranean origin for the species.

**Conclusions:**

In our study *Petrosia ficiformis* showed extreme levels of inbreeding and population differentiation, which could all be linked to the poor swimming abilities of the larva. However, the observed moderate migration patterns are highly difficult to reconcile with such poor larval dispersal, and suggest that, although unlikely, dispersal may also be achieved in the gamete phase. Overall, because of the high genetic diversity in the Eastern Mediterranean and frequent mass mortalities in the Western Mediterranean, we suggest that conservation efforts should be carried out specifically in those areas of the Mediterranean to safeguard the genetic diversity of the species.

**Electronic supplementary material:**

The online version of this article (10.1186/s12862-018-1343-6) contains supplementary material, which is available to authorized users.

## Background

The Mediterranean basin is one of the most diverse places on Earth in terms of animal and plant diversity, yet one of the most threatened (e.g., [1, 2]. Most of these threats are human-related and comprise pollution, direct and indirect mechanical damage and extraction of species, which result in habitat degradation, fragmentation, and loss, and alterations to the native biodiversity, among others [1, 2]. According to Coll and collaborators [2], several areas in the Mediterranean, such as the North Adriatic Sea, the Spanish coasts, and the Gulf of Lion in France, are known to have high diversity in terms of marine invertebrates (at least those with commercial value). These diversity hotspots are also the most threatened, which presents a dramatic scenario for the conservation of invertebrates in this area of the planet.

Most notably, climate-related natural disasters are considered one of the acutest threats for invertebrates in the Mediterranean Sea, with the Eastern Mediterranean being the most affected region [2–4]. Although the conservation emphasis is often on commercially important species or those considered as threatened in the Red List [5], a wide variety of sessile invertebrates are strongly affected by temperature anomalies, pollutants or its combined effect [3, 6]. In particular, sponges of the order Dictyoceratida have been considered one of the most affected invertebrates by warming oceans [3, 7–9]. Other than dictyoceratids, the emblematic haplosclerid sponge *Petrosia* (*Petrosia*) *ficiformis* (Poiret, 1789) has been recurrently reported to be affected by necrotic processes that decimated their populations in several areas of the Mediterranean [3, 10]. In all cases, mortalities were linked to dramatic increase in seawater temperature and heavier rainfall in the area [10]. The necrotic white tissue observed in Ligurian and Spanish populations of *P. ficiformis* was attributed to the loss of cyanobacterial symbionts and a heat shock destabilizing the adhesion and cytoskeletal organization of the exopinacoderm [10]. A similar situation was described when corals bleach due to the loss of zooxanthellae [11].

*Petrosia ficiformis* was originally described from what was then called the Berber coast (now Morocco, Algeria, Tunisia and Libya). It is a common and ubiquitous shallow water species [12], distributed along the entire Mediterranean Sea and the Macaronesia (Azores, Madeira, and Canary Islands) [13]. *Petrosia ficiformis* is a massive or lobate wine-colored sponge that grows slowly (approximately 1% monthly increase in volume) [14]. Besides being a frequent component of benthic assemblages in the Atlanto-Meditterranean shallow waters (10–50 m depth) and a structural species, it has economic value because of its ability to produce bioactive products [12]. *Petrosia ficiformis* is an oviparous species, which exhibits a special type of ciliated crawling stereoblastula [15] with very limited dispersal potential. These features, slow growth and restricted dispersal, make *P. ficiformis* a highly vulnerable species with a poor ability to recovery during disease episodes.

The shallow waters of the Atlanto-Mediterranean region experience a range of climatic conditions, including subtropical, temperate and subarctic conditions, leading to a high diversity of marine invertebrates generally, and of sponges in particular [16]. This area has experienced major geological events such as the Messinian Salinity Crisis (MSC), approximately 5 Myr BP, with a massive drop in available coastal habitat [17]. In addition, sea surface temperature and sea level drastically decreased during the Last Glacial Maximum (LGM, 30–19 Myr BP). All these geological events have profoundly shaped the marine fauna diversity patterns [16, 18, 19], and in particular, the present-day sponge fauna in the Mediterranean Sea is suggested to be the result of a recolonization from Atlantic refugia (Ibero-Moroccan and West African areas) after the MSC [20, 21]. More recently, during the interglacial periods of the Pleistocene, the warmer Mediterranean Sea received colder shallow Atlantic surface water, and the reverse occurred during glacial periods [17, 18]. In this sense, the Alboran Sea is considered to be an important area containing information on the past and recent faunistic interchange between the Atlantic and the Mediterranean Sea [18, 19]. According to Patarnello and collaborators [22], two plausible scenarios can be drawn for species spanning their distributions in the Mediterranean Sea and the Atlantic Ocean: 1) “complete genetic separation between Atlantic–Mediterranean populations since the early Pliocene”, and 2) “complete absence of population differentiation, usually following late Pleistocene recolonization”.

Population genetic analyses of marine Atlanto-Mediterranean invertebrate species have been performed for some taxa, including echinoderms [23–28], molluscs (e.g., [29, 30]), arthropods (e.g., [31, 32]), and sponges (e.g., [33–36]). In general, for most marine invertebrates, migration of Atlantic populations into the Mediterranean is lower than between the Eastern and Western Mediterranean basins mostly due to the presence of the Almeria-Oran front (e.g. [26, 30]). Also, higher local population differentiation is observed for sessile invertebrates with short larval duration phases, while the genetic structure of mobile invertebrates with long larval duration phases seems to be largely affected by a strongest effect of oceanographic fronts [37]. However, the genetic structure and connectivity patterns of sponges, covering both the Eastern and Western Mediterranean sub-basins and the Northeastern Atlantic, have been studied in few species, mostly with mitochondrial markers with low variability, providing poor resolution to infer detailed patterns [38, 39] or are based on limited sampling in the Atlantic region [34, 35]. Interestingly, the phylogeographic patterns inferred for Atlanto-Mediterreanean sponges appear to be the result of contrasting past demographic events. While *Crambe crambe* (Schmidt, 1862) is a Mediterranean species that later colonized Macaronesia via human transport [38], *Phorbas fictitius* (Bowerbank, 1866) originated in the Macaronesian archipelagos, colonizing the mainland using currents that flow towards the Iberian Peninsula [39].

**Fig. 1:**
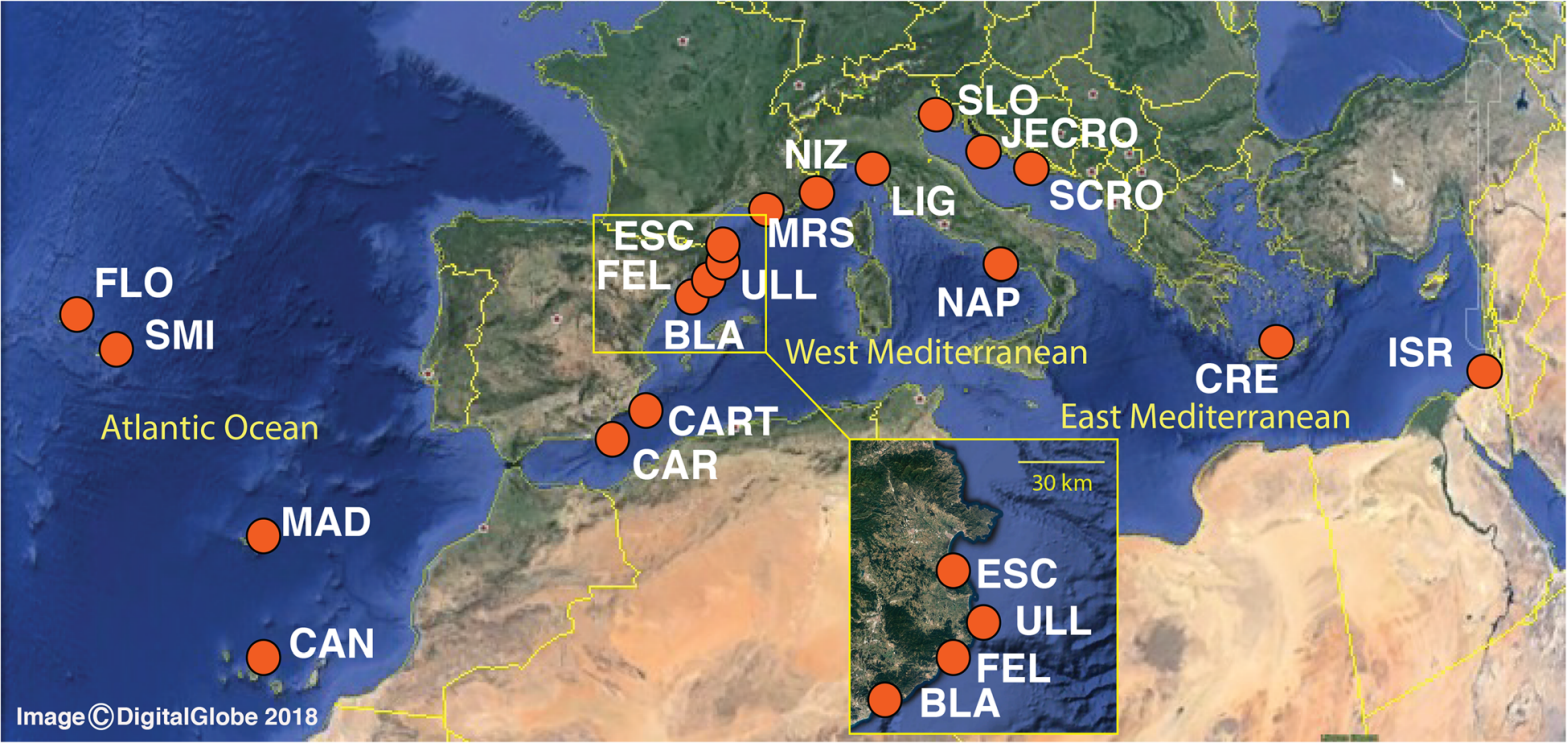
Sampling sites spanning the entire distribution range of *Petrosia ficiformis*. Maps were obtained and modified from Google maps (Map data: Google, DigitalGlobe, 2018)

The main goal of this study was to evaluate the distribution of the genetic diversity and assess population differentiation and connectivity of *P. ficiformis* in its current distribution range (Fig. 1 and Table 1), including areas where massive mortalities have been reported, using highly informative microsatellite markers. In addition, our aim was to infer the phylogeographic history of the species to understand recent demographic events and establish its potential geographical origin.

**Table 1:** Details on collection sites and number of individuals per location for *Petrosia ficiformis*

Location	Code	N	Coordinates	Region
Sao Miguel Is., Azores, Portugal	SMI	16	37.731764, −25.60182	A, Az, M
Flores Island, Azores, Portugal	FLO	10	39.503603, −31.243692	A, Az, M
Madeira, Portugal	MAD	14	32.701961, −16.758297	A, M, SM
Tenerife,Canary Islands, Spain	CAN	8	28.367267, −16.343625	A, M, SM
Carboneras, Spain	CAR	27	36.993139, −1.887294	WM, PA
Cartagena, Cabo de Palos, Spain	CART	7	37.629778, −0.688783	WM
Blanes, Spain	BLA	27	41.673213, 2.802638	WM
Els Ullastres, Llafranç, Spain	ULL	18	41.886108, 3.195997	WM
Sant Feliu de Guíxols, Spain	FEL	12	41.770931, 3.030411	WM
L’Escala, Spain	ESC	10	42.120158, 3.139733	WM
Grotte à Corail, Marseille, France	MRS	12	43.210339, 5.332714	WM
Nice, France	NIZ	16	43.682825, 7.320811	WM
Portofino, Liguria, Italy	LIG	16	44.305681, 9.214097	WM
Bacoli, Naples, Italy	NAP	16	40.780506, 14.083178	WM
Trieste, Slovenia	SLO	13	45.688828, 13.639978	EM, AS
South Adriatic (Croatia)	SCRO	14	43.547203, 15.872928	EM, AS
Jelsa (Hvar Island) Croatia	JECRO	14	43.176067, 16.696278	EM, AS
Crete, Greece	CRE	10	35.488389, 24.155528	EM
Achziv, Israel	ISR	20	33.051517, 35.101528	EM
TOTAL		280		

*Abbreviations*: *A* Atlantic, *AS* Adriatic Sea, *Az* Azores, *EM* Eastern Mediterranean, *M* Macaronesia, *PA* Pre-Alboran, *SM* Southern Macaronesia, *WM* Western Mediterranean

## Results

### Genetic diversity in *Petrosia ficiformis*

The average number of alleles per site was 37.05, ranging from 24 in FLO, LIG, NAP, SLO, SCRO, JECRO, CRE, and ISR to 81 in BLA (Table 2, Additional files 1, 2). However, the number of effective alleles (Table 2) was highest in ISR (4.276) and lowest in FLO (1.532), similar to what we observed for the allelic richness following rarefaction (Table 2, Additional file 1). The allele frequency per locus and population is shown in Additional file 2. Only four individuals were identified as potential clones with GENODIVE: two in CAR and two in ULL, although one of the two potential clones observed in CAR was not genotyped for one microsatellite marker (30PETRO).

**Table 2:** Results of the bottleneck analysis and effective population size for the 19 populations of *Petrosia ficiformis*

Pop.	ϴ	CI 95%	A	Ae	rA	Ho	He	FIS	HWE	Wilcoxon rank test	
										(2t_SMM)	(2t_TPM)
SMI	0.003	0.0003–0.0048	30	1.938	2.567	0.383	0.403	0.089	**	0.426	0.820
FLO	0.020	0.0054–0.0228	24	1.532	2.123	0.307	0.341	0.156	**	0.301	0.570
MAD	0.085	0.0800–0.0953	38	2.322	3.019	0.393	0.48	0.217	***	0.160	0.922
CAN	0.050	0.036–0.0530	41	3.016	3.888	0.636	0.618	0.045	**	0.432	0.160
CAR	0.084	0.0710–0.0970	49	2.694	3.361	0.579	0.54	−0.052	ns	**0.024**	0.625
CART	0.092	0.0803–0.0980	47	2.707	4.347	0.586	0.581	0.068	ns	**0.014**	0.105
BLA	0.093	0.0884–0.0980	81	3.901	4.847	0.681	0.718	0.071	***	**0.001**	0.084
ULL	0.085	0.0800–0.0976	37	2.233	2.855	0.613	0.503	−0.191	**	0.625	0.625
FEL	0.036	0.0444–0.0601	51	3.262	3.997	0.658	0.624	−0.01	ns	0.625	0.625
ESC	0.003	0.0000–0.0150	40	2.732	3.552	0.627	0.56	−0.065	ns	1.000	0.625
MRS	0.036	0.0102–0.0174	50	2.62	3.922	0.489	0.56	0.173	***	**0.003**	**0.010**
NIZ	0.040	0.0438–0.0562	48	2.478	3.563	0.551	0.562	0.057	***	**0.005**	**0.019**
LIG	0.018	0.0002–0.0274	60	3.964	4.736	0.509	0.706	0.311	***	0.922	0.193
NAP	0.061	0.0498–0.0845	53	3.342	4.145	0.677	0.682	0.042	ns	0.625	0.032
SLO	0.090	0.0832–0.1000	41	2.919	3.567	0.7	0.633	−0.065	ns	0.322	**0.014**
SCRO	0.029	0.0060–0.0223	53	2.775	3.804	0.574	0.553	−0.001	ns	**0.007**	0.105
JECRO	0.096	0.0854–0.1000	55	2.96	4.181	0.548	0.617	0.151	***	**0.001**	0.084
CRE	0.007	0.0000–0.0133	48	2.822	3.926	0.627	0.556	−0.075	ns	**0.014**	0.131
ISR	0.007	0.0007–0.0133	77	4.276	4.97	0.778	0.738	−0.028	ns	**0.010**	1.000

*Abbreviations*: *Pop* population, ϴ, Theta; 25–75% confidence intervals for ϴ, *Ns* number of individuals, *A* number of alleles, *Ae* Number of effective alleles, *rA* number of alleles after rarefaction, *Ho* observed heterozygosity, *He* expected heterozygosity, *F*_*IS*_ inbreeding coefficient, *HWE* deviation from Hardy-Weinberg equilibrium, *ns* not significant; * = *p* < 0.05; ** = *p* < 0.01; *** = *p* < 0.001; TPM, two-phase model; SMM, stepwise-mutation model

**Fig. 2:**
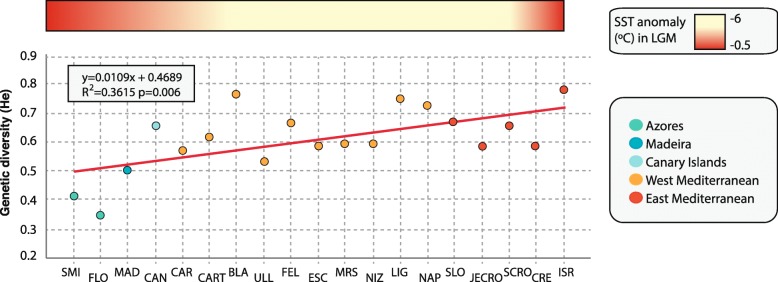
Correlation between genetic diversity (He) of *Petrosia ficiformis* and geographical location. The sea surface temperature (SST) anomalies during the last glacial maximum (30,000–19,000 years ago) is plotted onto the graph. Data for SST was obtained from [79]

Genetic diversity was highest in the Eastern Mediterranean site of ISR (H_e_ = 0.738) and the Western Mediterranean sites of BLA (H_e_ = 0.718), LIG (H_e_ = 0.706), and NAP (H_e_ = 0.682) (Table 2, Fig. 2, and Additional file 1). In the Atlantic, CAN showed the highest genetic diversity (H_e_ = 0.618), while the rest of sites showed values below 0.500 (Table 2, Fig. 2, and Additional file 1). The lowest genetic diversity value was detected in FLO (H_e_ = 0.341) (Table 2, Fig. 2, and Additional file 1). The average genetic diversity (H_e_) for the 19 populations was 0.578. Within the Mediterranean populations, observed heterozygosity values (H_o_) were higher in ISR, SLO, NAP and BLA, while in the Atlantic populations, CAN showed the highest H_o_ (Table 2 and Additional file 1). Significant differences in H_e_ values were detected in the comparisons including Atlantic populations (Atlantic vs. Western Mediterranean: *p*-value = 0.00482; Atlantic vs. Eastern-Mediterranean: *p*-value = 0.0014) but not in the comparisons between Western and Eastern-Mediterranean (*p*-value = 0.1133).

The inbreeding coefficients (F_IS_) were positive in 11 out of the 19 sites (SMI, FLO, MAD, CAN, CART, BLA, MRS, NIZ, NAP, LIG, and JECRO), LIG being the site with the highest value (F_IS_ = 0.311; Table 2, Additional file 1), indicating that individuals in these sites were more related than expected if random mating occurs. The closest value to perfect HWE (F_IS_ = 0) was detected in SCRO (F_IS_ = − 0.001; Table 2, Additional file 1), indicating that individuals within this site were less related than expected, and other populations (ULL, ISR, CRE, SLO, ESC, FEL, and CAR) also showed negative (then effectively 0) values for F_IS_. The populations of CAR, CART, FEL, ESC, NAP, SLO, SCRO, CRE and ISR were in HWE (Table 2, Additional file 1). The microsatellite markers that contributed the most to any HWE deviation in the rest of populations were 1PETRO, 17PETRO and 19PETRO (Table 2, Additional file 1), but excluding these three markers did not change the result, and therefore we included them in all subsequent analyses.

### Population differentiation in *Petrosia ficiformis*

**Fig. 3:**
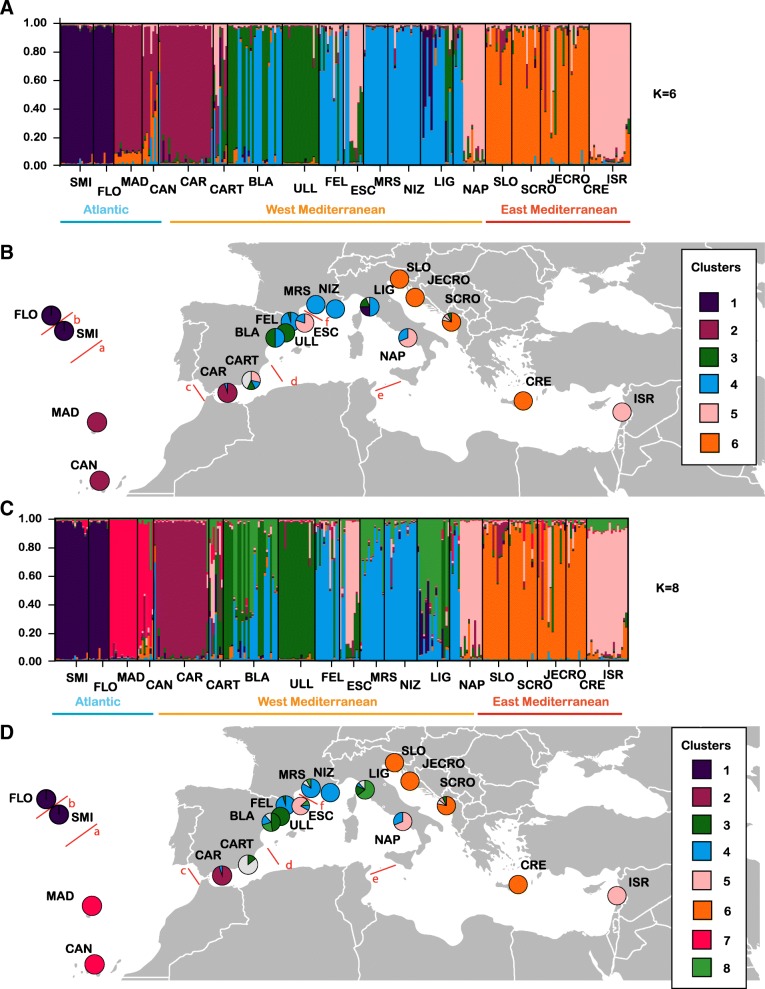
Individual genotype assignment of *Petrosia ficiformis* to clusters (K) as inferred by STRUCTURE for all studied sites with **a–b**. K = 6 and **c–d**. K = 10. In **b** and **d**, pie frequency charts depict the percentage of individuals assigned to each of the 5 clusters from **b** and **c** for all sites. Colors are assigned as in **b** and **d** except grey which shows the percentage of individuals for which the assignation was not clear (Q-value cutoff 60). Red lines indicate barriers detected by BARRIER ranked *a* to *f* in order of importance. Maps were modified from Wikipedia under a Creative Commons license (User Canuckguy)

The optimal number of clusters identified using delta K (Additional file 3A) for *P. ficiformis* was six, then eight, and finally ten (Fig. 3, Additional file 3A–B). In the optimal scenario considering K = 6, the following clusters were obtained (Fig. 3a–b): Cluster 1 (purple): grouping the populations in Azores (SMI, FLO); Cluster 2 (crimson): grouping the other populations in Southern Macaronesia (MAD, CAN) and the south of Spain (CAR); Cluster 3 (green): containing ULL and half of the population of BLA, as well as some individuals from the populations CART, FEL, LIG, and SCRO; Cluster 4 (blue): containing the rest of Western Mediterranean populations (half of the population of BLA, most FEL, some individuals in ESC, MRS, NIZ, half LIG, and some individuals in NAP); Cluster 5 (pink): comprised of some individuals in CART, most ESC individuals, half of the NAP population, and the Eastern Mediterranean population of ISR; and finally Cluster 6 (orange): the Adriatic populations (SLO, SCRO, JECRO) and the Eastern Mediterranean population of CRE. The second optimal clustering identified 8 clusters (Fig. 3c–d), detecting substructure in Cluster 2 (Fig. 3c), with the Southern Macaronesia populations and CAR appearing in two different clusters (clusters 2 and 7) and a new cluster (cluster 8) grouping individuals from BLA, ESC, MRS, and LIG (Fig. 3c–d). The intriguing cluster 5 from K = 6 and K = 8, grouping individuals from quite disparate populations (ESC, NAP and ISR), was not detected in the scenario with K = 10, showing separation of those populations in two different clusters (Additional file 3B). Using a multivariate approach, the population structure and individual assignment to a given population assessed with *adegenet* revealed that 6 to 10 clusters would define the most probable structure (Additional file 3C), the 10-cluster hypothesis having the lowest BIC (Additional file 3D). In the 10-cluster scenario (Additional file 3D), most individual assignments were similar to those obtained by STRUCTURE (Fig. 3c-d and Additional file 3D), except for those of CAN, CAR, and SLO, which were assigned here to separate clusters (Additional file 3D).

**Fig. 4:**
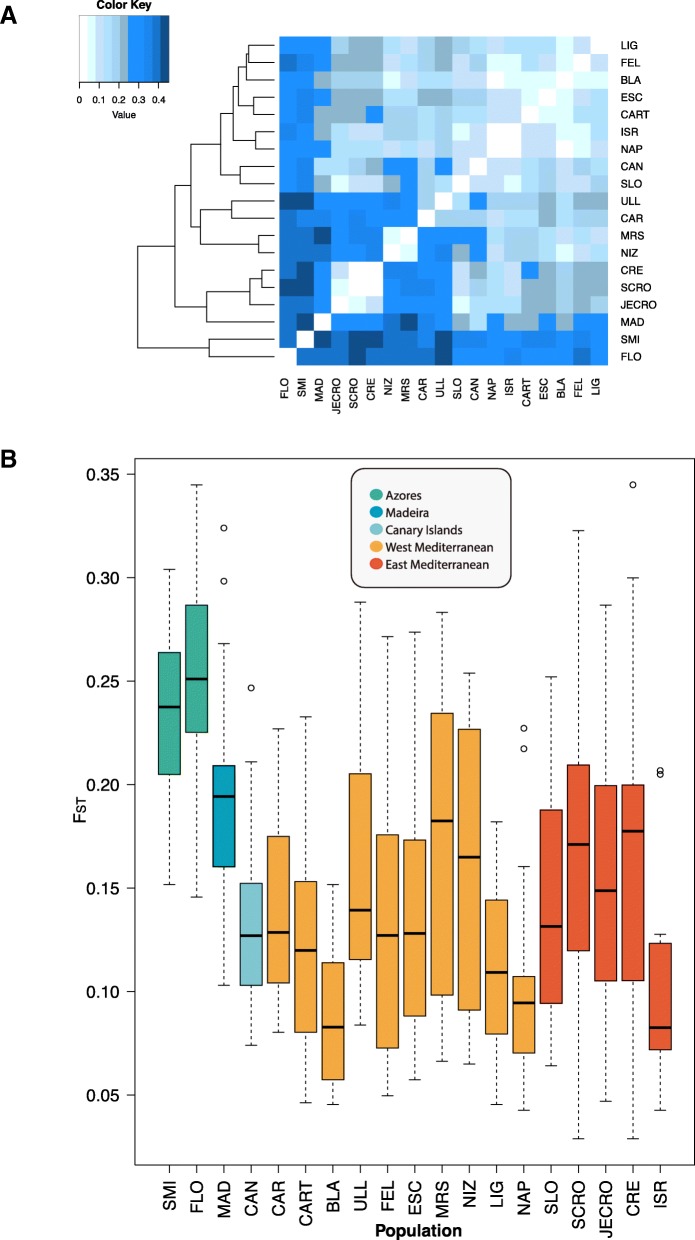
**a**. *F*_ST_ comparisons between 19 populations of *Petrosia ficiformis*. All comparisons showed significant *p*-values except for the comparison between CRE and SCRO. Actual values for each comparison can be found in Additional file 2. **b**. Average values (and standard errors) of *F*_*ST*_ for each sampling site across *Petrosia ficiformis* distribution

Population differentiation was also assessed using *F*_*ST*_ pairwise comparisons, revealing significant differences among all population pairs (*p* < 0.008), except for the population pairs CRE and SCRO (Fig. 4a and Additional file 4). The highest *F*_*ST*_ values (> 0.400) were observed between Atlantic sites and Western and Eastern Mediterranean sites: first between SMI and the following sites: (1) MAD, ULL, SCRO, and CRE; (2) between FLO and ULL, MRS, SCRO, CRE, and (3) between MRS and MAD (Fig. 4a and Additional file 4). The lowest value of *F*_*ST*_ (0.02233) was observed between SCRO and CRE (Additional file 4). The global *F*_*ST*_ for all populations was 0.2348 and the average values were around 0.15 (Fig. 4b).

To test whether the population differentiation observed with the *F*_*ST*_ pairwise analyses was due to an isolation by distance (IBD), a Mantel test was performed for the whole dataset, and approximately 47% of the variation observed could be attributable to IBD (*r*^*2*^ = 0.466, *p* = 0.03). However, due to the limitations of the Mantel test [40, 41], we also performed stratified Mantel tests within each of the clusters separated by genetic breaks identified by BARRIER (see below), finding no significant IBD in any of them (Clusters tested: a: CAN, MAD, CAR: *r*^*2*^ = 0.615, *p* = 0.310; b: Western Mediterranean 3: *r*^*2*^ = − 0.168, *p* = 0.307; c: Eastern Mediterranean: *r*^*2*^ = 0.245, *p* = 0.173).

BARRIER located six a priori selected barriers in order of importance: *a*, separating the Azores sites; *b*, between SMI and FLO; *c*, separating the Atlantic sites from the Mediterranean ones; *d*, located approximately at the Almeria-Oran front and separating CAR and CART from the rest of the Mediterranean sites; *e*, located approximately at the Strait of Sicily front separating the Western and Eastern Mediterranean sites; and finally *f*, isolating the Spanish sites (BLA, FEL, ULL, and ESC) from the rest of the Mediterranean sites (Fig. 3b, d).

**Fig. 5:**
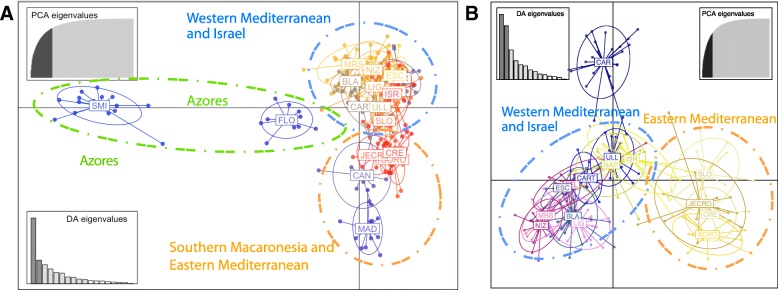
Population differentiation in *Petrosia ficiformis.* **a**. Subdivision of all sites according to DAPC analysis. **b**. Subdivision of the Mediterranean sites according to DAPC analysis

The DAPC analyses for all the sampling sites and only the Mediterranean sites (Fig. 5a–b) rendered different patterns. When the matrix containing all the sampling sites was analysed, the Azores populations (SMI and FLO) were clearly separated from the rest considering both axes (Fig. 5a) and the Y-axis also separated the populations of the Eastern Mediterranean + Southern Macaronesia (MAD, CAN) and those in the Western Mediterranean and ISR (Fig. 5a). When only the Mediterranean sites were analysed (Fig. 5b), a clear separation of the Eastern and Western Mediterranean sites was observed, with only the CAR population (named Pre-Alboran site because it is located before the Almeria-Oran Front) separated from the rest of Mediterranean sites. It is important to note that in these two cases, the ISR site was closer to the Western Mediterranean sites than to the Eastern sites (Fig. 5a–b).

All the AMOVA analyses (A. Atlantic vs. Mediterranean; B. Atlantic vs. Western Mediterranean vs. Eastern Mediterranean; and C. Azores vs. Southern Macaronesia vs. Western Mediterranean vs. Eastern Mediterranean) were significant among groups, populations and individuals (Table 3). However, the analysis that rendered the highest value for the percentage of variance explained among the groups used (13.2%) was that using the groups Azores, Southern Macaronesia, Western Mediterranean, and Eastern Mediterranean (Table 3).

**Table 3:** Results of the AMOVA performed using three different groupings for the investigated populations of *Petrosia ficiformis*: A. Atlantic (SMI, FLO, CAN, and MAD) vs. Mediterranean (CAR, CART, BLA, ULL, FEL, ESC, MRS, NIZ, LIG, NAP, SLO, SCRO, JECRO, CRE, and ISR); B. Atlantic (SMI, FLO, CAN, and MAD) vs. Western Mediterranean (CAR, CART, BLA, ULL, FEL, ESC, MRS, NIZ, LIG, and NAP) vs. Eastern Mediterranean (SLO, SCRO, JECRO, CRE, and ISR); and C. Atlantic (SMI and FLO) vs Macaronesia (CAN and MAD) vs. Western Mediterranean (CAR, CART, BLA, ULL, FEL, ESC, MRS, NIZ, LIG, and NAP) vs. Eastern Mediterranean (SLO, SCRO, JECRO, CRE, and ISR). Atlantic See Table 1 for full name of locations. Significant *p-*values appear in bold letters

A. Atlantic vs. Mediterranean						
Source of Variation	%var	*F*-value	Std.Dev.	c.i.2.5%	c.i.97.5%	*p*-value
Within Individuals	0.730	0.270	0.068	0.134	0.385	–
Among Individual	0.030	0.039	0.081	−0.122	0.180	**0.003**
Among Populations	0.194	0.204	0.017	0.169	0.233	**0.001**
Among Groups	0.046	0.046	0.011	0.026	0.069	**0.006**
B. Atlantic vs. Western Mediterranean vs. Eastern Mediterranean						
Source of Variation	%var	*F*-value	Std.Dev.	c.i.2.5%	c.i.97.5%	*p*-value
Within Individuals	0.722	0.278	0.069	0.144	0.393	–
Among Individuals	0.033	0.043	0.082	−0.112	0.183	**0.001**
Among Populations	0.152	0.167	0.014	0.140	0.192	**0.001**
Among Groups	0.094	0.094	0.025	0.052	0.145	**0.001**
C. Azores vs. Macaronesia vs. Western Mediterranean vs. Eastern Mediterranean						
Source of Variation	%var	*F*-value	Std.Dev.	c.i.2.5%	c.i.97.5%	*p*-value
Within Individuals	0.714	0.286	0.069	0.145	0.402	–
Among Individuals	0.030	0.040	0.081	−0.119	0.183	**0.001**
Among Populations	0.124	0.143	0.012	0.119	0.165	**0.001**
Among Groups	0.132	0.132	0.019	0.100	0.171	**0.001**

### Migration patterns in *Petrosia ficiformis*

**Fig. 6:**
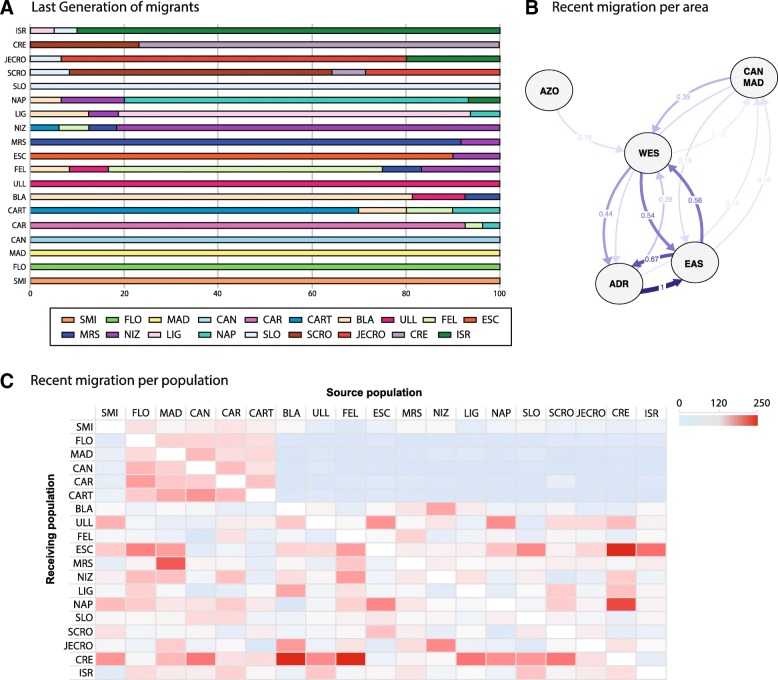
Migration patterns in *Petrosia ficiformis.* **a**. Number of last generation migrants indicated in percentage of the population for each sampling site. **b**. Migration estimates among the four areas considered AZO (SMI, FLO), CAN, MAD, WES (CAR, CART, BLA, ULL, FEL, ESC, MRS, NIZ, LIG, and NAP), and EAS (SLO, SCRO, JECRO, CRE, and ISR). Only values over 0.1 are shown in the graph. **c**. Heatmap of coalescent migration estimations. The scale refers to migration rates between 0 and 250 (actual data can be seen in Additional file 5)

We identified only 7 individuals as last generation migrants: one from FEL into CAR, another one from FEL into CART, one from MRS into BLA, one from ULL into FEL, two from NIZ into ESC and LIG, and one from NAP into LIG, with mean distances among these populations ranging from 43 to 632 km. The migration patterns obtained with GENODIVE showed that Atlantic sites did not contain any migrant among their individuals (Fig. 6a). In the Mediterranean, the different sites showed a percentage of migrants between 3.7% (from FEL in CAR) and 28% (from CRE in SCRO), except for SLO, where we did not observe any (Fig. 6a). Interestingly, when analyzing the directionality of recent migration events among areas, the main gene flow appeared between the Adriatic Sea and the Eastern Mediterranean basin in both directions (Fig. 6b), and then from the Eastern Mediterranean into the Western Mediterranean and vice versa (Fig. 6b). This analysis also indicates that there were low levels of migration from the Atlantic populations of MAD and CAN (MAC) into the Western Mediterranean (Fig. 6b), and almost negligible migration from the Azores to the Western Mediterranean (Fig. 6b). When the migration patterns were analysed in more detail using pairwise comparisons among all populations (Fig. 6c and Additional file 5), it was clear that the gene flow was relatively high among the Macaronesian islands (SMI, FLO, MAD, and CAN) and the Southern Spain populations (CAR and CART) and also with CRE, and moderate with the rest of the Mediterranean, but no gene flow was detected from the Mediterranean into the Atlantic populations. Within the Mediterranean, we detected high migration rates among the populations in the Western Mediterranean and also with the Aegean Sea, and from the Adriatic into ESC and NAP (Fig. 6c and Additional file 5). The highest effective population sizes, assessed from theta, were found for some populations from the Atlantic, Western Mediterranean and the Adriatic: FLO, MAD, CAN, CAR, CART, BLA, ULL, SLO and JECRO (Table 2).

### Demographic events in *Petrosia ficiformis*

Several populations showed signs of a recent reduction of their population size using the Wilcoxon rank tests under two different models: with SMM, the populations of CAR, CART, BLA, MRS, NIZ, SCRO, JECRO, and ISR appeared to have a bottleneck, but bottlenecks in only MRS, NIZ, and SLO were detected under the TPM model (Table 2). In any case, our results should be taken with caution since our sample numbers were sometimes too low to robustly detect bottleneck events.

## Discussion

### Negligible clonality in populations of *Petrosia ficiformis*

Sponges are commonly considered clonal organisms since they are capable of both sexual and asexual reproduction, the latter including gemmulation, fission, and budding [42]. The larvae of many clonal organisms, not only sponges but also of bryozoans and cnidarians, are usually short-lived small propagules that remain within close proximity of their progenitors [43]. Therefore, other dispersal mechanisms maybe responsible for the large distribution ranges observed in many clonal organisms, including rafting and passive dispersal of newly settlers by oceanic currents [43]. However, even though that might be true for some sponges (e.g., [44]), *Petrosia ficiformis* seems to not rely on any dispersal mechanism via asexual reproduction. In our analysis, only two out of 280 individuals (0.7%) showed potential clonality signatures. This almost negligible clonality contrasts with the high values obtained for other sponges [45] using similar sampling strategies and geographic separation of locations, but could be easily explained by their different biological traits. *Petrosia ficiformis* is a massive sponge, with no asexual reproduction documented so far [15]. In contrast, both *C. crambe* and *Cliona* spp. are encrusting sponges known to undergo frequent fission processes [46, 47], which explains their high rates of clonality. In our case, it seems that the connectivity and gene flow between populations of *P. ficiformis* is mostly accomplished by sexual reproductive strategies since no asexual reproduction or any other dispersal strategy (e.g., rafting) have ever been reported for *P. ficiformis*.

### Contrasting patterns in inter- and intrapopulation structure along the distribution range of *Petrosia ficiformis*

The average genetic diversity (He) of *P. ficiformis* (0.578), falls into the range (0.4–0.9) observed using microsatellite markers in other Mediterranean sponges, including poecilosclerids, dictyoceratids, and scopalinids [33–36, 48], as well as in clionaids, petrosiids and calcareous sponges in other latitudes (e.g., [45, 49–51]). Interestingly, relatively similar (although slightly higher) values of genetic diversity (He) to those of *P. ficiformis* are usually reported for other Mediterranean sessile (e.g., ~ 0.8 in *Corallium rubrum*, [52]; ~ 0.7 in *Paramuricea clavata*, [53]) and vagile organisms (e.g., ~ 0.6 in the bluefin tuna, [54]; ~ 0.75 in the sea bass, [55]; ~ 0.8 in *Tripterygion delaisi*, [56], ~ 0.8 in *Paracentrotus lividus*, [57]; ~ 0.7 in *Echinaster sepositus,* [26]).

The departures from HWE in 10 out of the 19 populations of *P. ficiformis* analysed (Table 2, Additional file 1) can be explained by several factors. In the Macaronesian archipelagos (except in CAN), the Western Mediterranean populations of BLA, MRS, NIZ, and LIG, and the Eastern Mediterranean population of JECRO, heterozygote deficiencies (and therefore inbreeding) seemed to be responsible. For many other organisms, the presence of null alleles, Wahlund effect [58], and the production of high numbers of sperm cells in free-spawners are also responsible for HW disequilibrium patterns [59]. For *P. ficiformis*, the incidence of null alleles was negligible in all populations (only one microsatellite showed null alleles), and no substructure attributable to Wahlund effect was detected in the population differentiation analyses. Other factors related to the sexual reproductive strategy of *P. ficiformis* might be playing a significant role in the departures for HWE observed here. Most sponge larvae have very limited dispersal capabilities, despite being free-swimming larvae [60–62], but *P. ficiformis* falls into the extreme end of the spectrum. In theory, the average distance of dispersal should be greater in oviparous species than in viviparous species, because it inevitably includes external fertilization [43]. However, even though *P. ficiformis* is an oviparous species, its larva has very peculiar features that restrict its dispersal capabilities compared to other sponge larvae. Instead of swimming in the water, they crawl for short periods (up to 2 weeks) until settlement [15]. Therefore, its dispersal capabilities are strongly limited by the slow crawling of the larvae. In addition, *P. ficiformis* is a gonochoristic species, and it devotes almost all its internal tissues to massive oocyte and sperm production [15]. But although large numbers of gametes are released in the water that could potentially disperse farther, the incrementing dilution factor favours fertilization within few meters in experiments performed in the field for marine invertebrates. Indeed, fertilization rates drop to less than 5% when gametes are dispersed to 1 m in sea urchins [63], and to 0% at 7 m in hydroids [64], although some species could still fertilize within 10 m [65]. In fact, using genetic markers in corals, the distance that sperm dispersed and mantained fertilization success was always less than 20 m [66]. However, a long lifespan (16–26 h) for the sperm of the ascidian *Botryllus schlosseri* [67] has been reported, and a similar phenomenon may exist in *P. ficiformis*. In all, inbreeding and population self-recruitment seem the most plausible causes of homozygote excess in *P. ficiformis*.

Only two populations that were not in HWE showed excess of heterozygotes (CAN and ULL). Whereas in studies with single nucleotide polymorphisms, gain of heterozygosis in populations not in HWE were mainly caused by genotyping errors [68], biological strategies such as selection against homozygotes, or extreme longevity of individuals were suggested for excess of heterozygotes in a sympatric Mediterranean sponge [33].

### Molecular signatures of demographic contractions in *Petrosia ficiformis*

Garrabou and collaborators documented die-offs of *Petrosia ficiformis* populations due to a disease outbreak in 2003 in the Provence Coast and the Gulf of Genoa [3], necrotic events in the Gulf of Genoa have also been reported in previous years [10]. In our study, the populations of MRS and NIZ had signatures of bottlenecks under different models (Table 2). But although BLA also presented a bottleneck, no similar die-offs were documented for the Catalan coast. However, during our periodic surveys conducted in 2004–2007 to establish the reproductive patterns of *P. ficiformis* in the area, we recurrently observed necrotic processes after egg spawning in February–March (Riesgo personal observations). Even though we did not record dead sponges, such necrotic processes could have led to die offs in other years in these populations.

In addition, although the sampling sites of SLO, SCRO, JECRO, CRE, and ISR also showed signatures of bottleneck in one test, massive population die-offs were never reported for those areas. Interestingly, while JECRO showed heterozygosity deficiency, SLO, SCRO, and ISR were in HWE (Table 2). In this case, the results of the bottleneck analysis should be taken with caution given the small sample sizes analysed, which could explain the incongruence between levels of allelic richness and heterozygosity in these populations.

### Population differentiation and connectivity in *Petrosia ficiformis*

We found strong population structure in our dataset both at small (i.e. significant *F*_ST_ between nearby populations), medium (i.e. significant differences between populations within basins) and large (i.e. significant population differentiation and AMOVAs among basins) geographical scales. The global and average Fst values for *P. ficiformis* were in the upper extreme of the range for sponges [69]. In *P. ficiformis*, population differentiation was even detected between populations located less than 50 km apart, similar to what was reported for *I. fasciculata* [36] although the differentiation was much more conspicuous in *P. ficiformis*.

Both population clustering approaches employed here, situated the most probable number of clusters (or K) between 6 and 10, rendering very similar results. While in the sympatric sponge *Ircinia fasciculata* [36] two clusters mostly explained the distribution of the genetic diversity structure observed in the Mediterranean Sea, for *P. ficiformis* we found a much more structured scenario. This could be attributed to a combination of both the limited dispersal potential of *P. ficiformis* (discussed above) and the stronger effect of oceanographic barriers to gene flow among the populations analysed here. Although in *P. ficiformis* an IBD pattern was detected initially in this and previous studies [70], after performing stratified Mantel tests for each of the clustering groups, IBD was not evident. Similarly, many sponge species have been reported as not showing IBD [36].

Indeed, instead of IBD, it seems that oceanographic fronts play a more prominent role in the spatial distribution of neutral genetic diversity of marine organisms around the globe (e.g., [71, 72]), but especially in sessile organisms [69]. In the Mediterranean, the most prominent barriers to gene flow and well-known for their isolating effects of the marine fauna are: the Almeria-Oran front, the Sicily Channel and the Otranto Strait [22, 37, 73]. In our study, the two first barriers overlapped with genetic discontinuities. However, the main barrier to gene flow was not found in the Mediterranean, but between the Azores populations and the continental ones (Fig. 3b, d), possibly driven by depth (> 4000 m) or distance (> 2000 km), with lack of suitable habitat for *P. ficiformis* in intermediate waters. Water circulation between the continent and the Azores archipelago [74] does not favour migration between them [75], and deep water is likely a barrier to the shallow-dwelling *P. ficiformis* [71, 76], potentially separating Azores from the rest of the Macaronesia (Madeira and the Canary Islands). Within the Mediterranean Sea, we detected the usual barriers to gene flow (Almeria-Oran front and Otranto Channel) reported in many other marine organisms [22, 37, 73], but interestingly, we found a genetic discontinuity in the Gulf of Lion, separating the populations in the Catalan coast from those in the French coast (Fig. 3b, d). In this area, freshwater from the Rhône river and the strong cooling induced by the Mistral south of the Rhône delta generate a layer of cold and less saline water during winter and spring [77] that could act like a barrier for sponge larval dispersal.

In our study, the closest populations geographically (BLA, ULL, FEL y ESC) did not appear in the same genetic cluster, and showed diverging proportions of individuals of 3 (when k = 6) or 4 (when k = 8) clusters. Some degree of larval retention could play a role in the case of ULL. That site is located in a submarine pinnacle that goes down to 65 m and is separated few km from the coast. It was identified as a highly-isolated population in K = 10, which could partially explain the low allelic richness of the site. The rest of microscale patterns of differentiation observed here are difficult to explain. In other sessile organisms with low dispersal abilities such as gorgonians, microscale differentiation has been explained mostly by a higher degree of self-recruitment [78]. In our case, also local gyres or connectivity with other non-sampled populations located in the Balearic Islands, Corsica and Sardinia, could be behind such genetic differentiation patterns.

### Colonization patterns and migration in *Petrosia ficiformis*

Interestingly, the total number of alleles, regardless of the estimate used, and the genetic diversity (H_e_) were always highest in the Mediterranean populations and lowest in those of the Macaronesian region (Table 2, Fig. 2 and Additional file 1), suggesting a Mediterranean origin for the species and subsequent colonization of Atlantic sites. A similar pattern was observed for the dictyoceratid *Spongia officinalis* [34] and the poecilosclerid *Crambe crambe* [38], where a colonization of the Macaronesia from the Mediterranean was also suggested. Using other molecular markers, the origin of *P. ficiformis* was also hypothesized to be Mediterranean, the source population most likely being located in the Eastern Mediterranean, which suffered a less dramatic seawater temperature drop (Fig. 2) during the LGM [70] (3 °C below current values [79]) and would likely have offered refuge as previously suitable habitat disappeared [22]. The affinity of *P. ficiformis* for warm waters is further supported by the fact that its reproductive season is triggered by the temperature rising in summer, and release of gametes with the abrupt temperature drop that characterizes the arrival of winter [36, 80].

The most recent calibrated phylogenetic tree for demosponges places the appearance of *Petrosia ficiformis* most likely during a period between the Cretaceous (approx. 100 Mya) and the Paleogene (23 Mya) [81], when the Mediterranean was still considered to be the Tethys Sea and harboured a highly-diverse warm-water biota [82]. Therefore, the appearance of *P. ficiformis* likely pre-dated the Messinian Salinity Crisis (MSC) during the Miocene (dated on 5.6 Mya, [17]), when several Mediterranean evaporitic basins could have acted as refugia for *P. ficiformis* [83]. Even though true marine conditions have been reported for some areas during the MSC [83], most areas show evidence of extensive desiccation and formation of hypersaline and freshwater sub-basins [84], but there is still controversy about the surviving fauna [85–87]. Alternatively, during the MSC, *P. ficiformis* could have survived in marine caves [82] where it is particularly abundant today. These scenarios are impossible to test with our dataset since the genomic traces of such events would not be detected with microsatellites (fast evolving markers), but certainly the higher levels of genetic diversity in the Eastern Mediterranean and the warm-water affinity of *P. ficiformis* point to this direction. The alternate possibility of *P. ficiformis* being an Atlantic species introduced in the Mediterranean after the Zanclean flood of the Mediterranean Sea, approximately 5.33 Mya [17], is not supported by the current lower genetic diversity and relative isolation of the Atlantic populations.

During the Pliocene, circulation patterns similar to the ones we currently find were established, preventing gene flow from the Mediterranean towards the Atlantic [17]. This was supported by our migration results showing null migration rates in that direction. On the other hand, our results suggest moderate gene flow between the populations of the Atlantic Ocean and those in the Alboran Sea, located closer to the Strait of Gibraltar, in both directions (Fig. 6), and moderate to low gene flow from the Atlantic towards the main Mediterranean basin (Fig. 6), except from the high values obtained from some Macaronesian populations into the Aegean Sea (Fig. 6c). The circulation patterns in the Mediterranean would have promoted connectivity from the Atlantic populations, along the southward coasts, and into the Aegean Sea (CRE), which could explain the high migration values detected. Currently, the strongest barrier to gene flow between the Atlantic Ocean and the Mediterranean is the Almeria-Oran front (see reviews of [22, 37]), an oceanographic barrier that has been shown to impair the connectivity of many benthic invertebrates (e.g., [25, 26, 88]), including sponges [35, 36]. Indeed, besides the genetic discontinuities found in the Macaronesian populations suggesting the presence of barriers to gene flow in the area, the Almeria-Oran front seems to be a prominent factor driving the current gene flow among the populations of *P. ficiformis* (Fig. 6c), preventing gene flow from the Atlantic into the Mediterranean. Moderate levels of migration were detected among the Mediterranean populations, with higher migration rates between the Adriatic and Eastern Mediterranean populations (Fig. 6c). In fact, all last generation migrants detected were exchanged among Mediterranean populations, although surprisingly among the Western ones, in a range from 40 to 630 km. This sort of direct migration is difficult to explain given the low dispersal abilities of the larva of *P. ficiformis*, and more research is definitely needed to understand the importance of gamete dispersal in the connectivity of this particular species. In this sense, a long gamete lifespan in *P. ficiformis*, as described in other organisms [67], could explain some of the dispersal necessary to cover such distances.

## Conclusions

Our study provides a detailed account of the distribution of genetic diversity across the geographic natural range of *P. ficiformis*, including information on how that diversity is connected and maintained during massive die-offs. In summary, it seems that the Eastern Mediterranean (Crete and Israel) harbours the greatest diversity, and we suggest the possibility that those could act as a reservoir in case population decimation affects other areas in the Mediterranean. However, that area shows higher levels of connectivity with the Adriatic Sea, and therefore in light of our results efforts should be made to protect populations of *P. ficiformis* not only in already existent Marine Protected Areas –MPAs– in the Eastern Mediterranean (or to create new MPAs to preserve them), but also in the Adriatic and Western Mediterranean in order to provide westward “gene flow corridors” for the species. In addition, the Atlantic populations deserve special attention, since they show the highest degree of reproductive isolation and if impacted, they will not receive recruits from closely related areas, which could end in their disappearance.

There are ongoing efforts to assess the impact of climate change on mass mortalities in the Mediterranean by modelling the current mass mortality risk associated with thermal stress of benthic coastal ecosystems [89]. However, those studies have only used one model species, *Paramuricea clavata*, for which genetic diversity and connectivity data are available (e.g., [53, 90]), and it is necessary to use more species in the models to obtain a more comprehensive assessment of the risks and impacts. Therefore, the information we provide in the present study is very much needed to design sound management and conservation strategies to mitigate the short term effects of global warming in the Mediterranean.

## Methods

A total of 280 sponges were sampled by the authors between 2008 and 2013 using SCUBA diving in 19 sites spanning the entire distribution range for *P. ficiformis*: the Atlanto-Mediterranean region that includes the Macaronesian islands of Azores, Madeira and the Canary Islands, and the entire Mediterranean Sea except for the Southern Mediterranean coastline (Fig. 1 and Table 1). Samples from some localities were collected by colleagues from their focal study areas (see acknowledgements). Variation in geographical distance among sampling sites ranged from 43 to 9115 km for the whole studied area. All sponges were collected at depths ranging from 15 to 35 m. Even though *Petrosia ficiformis* is a relatively common sponge in the Atlanto-Mediterranean, in some localities it was impossible to find more than 12 specimens. Tissue pieces of approximately 2 cm^3^ were excised with scissors/knives and preserved in 96% ethanol, replaced three times with fresh ethanol, and stored at − 20 °C. DNA was extracted with the DNeasy Blood & Tissue™ kit (Qiagen) following the manufacturer’s indications with minor modifications on the lysis time (performed overnight) and the elution step (performed twice using 50 μl of buffer EB each time).

### Microsatellite amplification and analysis

All 280 individuals were genotyped at 10 unlinked microsatellite loci (1PETRO, 4PETRO, 7PETRO, 11PETRO, 15PETRO, 17PETRO, 18PETRO, 19PETRO, 25PETRO, and 30PETRO) previously described [91], using the PCR conditions described therein. The sizes of the fluorescently labeled PCR products were estimated using an internal size marker (GeneScanTM 500 LIZ) on an ABI Prism™ 7700 Sequencer (Applied Biosystems) and analysed with Peak Scanner v1.0™ (Applied Biosystems). All populations (and therefore individuals) were randomly located in the 12 plates genotyped.

### Genetic diversity analyses in *Petrosia ficiformis*

GenAlEx 6.5 [92] was used for the estimations of the observed (H_o_) and expected (H_e_) heterozygosity, and the fixation index (F_IS_). Differences between H_e_ values among basins (Atlantic Ocean: FLO, SMI, MAD, CAN; Western Mediterranean: CAR, CART, BLA, ULL, FEL, MRS, NIZ, LIG, NAP, and Eastern Mediterranean: SLO, SCRO, JECRO, CRE, and ISR) were calculated using two-tailed *T* tests in Statplus 6 (AnalystSoft). In addition, Genepop on the web version 4.0.10 [93, 94] was used to obtain values for departure from Hardy Weinberg equilibrium (HWE) by locus and population using a probability test with level of significance and the following Markov chain parameters: 5000 dememorization steps, 1000 batches, and 5000 iterations per batch. Other measures of genetic diversity, such as the total number of alleles per locus and population, and number of effective alleles (=1/∑π^2^, where ∑π^2^ is the sum of the squared population allele frequencies) were calculated with GenAlEx and GENODIVE version 2.0b23 [95]. In order to correct for differences in sample sizes, the rarefaction method implemented in FSTAT 2.9.3.2 [96] was used to obtain the allelic richness at each locus. Clonality was assessed using GENODIVE and ignoring missing alleles.

### Population structure and differentiation in *Petrosia ficiformis*

Several methods to assess population structure and differentiation in *P. ficiformis* were used: two based on clustering approaches (STRUCTURE and a Discriminant Analysis of Principal Components, DAPC) and four distance-based methods: *F*_ST_ estimations, Isolation by Distance (IBD), BARRIER, and Analyses of the Molecular Variance (AMOVA).

The assignment of individuals to each population was performed using a Bayesian clustering approach in STRUCTURE 2.3.4 [97], that calculates population allele frequencies and then assigns individuals to populations probabilistically, always based on the estimates of Hardy Weinberg equilibrium (HWE) and/or linkage equilibrium. The specific parameters used were *admixture*, since although we had no previous knowledge on the origin of the populations studied, we assumed that a proportion of individuals can have recent ancestor coming from multiple populations, *no locprior*, since no additional sample-characteristic data was available, and *correlated allele frequencies*, because we had no previous knowledge on the correlation levels across populations [98]. The program was run with a burn-in time of 100,000 repetitions and 100,000 iterations (MCMC), setting the putative K (predicted number of genetic units) from 2 to 20 (one cluster more than the number of sampling sites considered in the analysis) and twenty replicated runs. The estimation of log probabilities of data Pr(X | K) for each value of K was evaluated by calculating ∆K, which accounts for the rate of change in the log probability of data between successive K values, currently considered a more reliable predictor of the true number of populations [99]. Convergence was assessed with the alpha parameter. Calculations and evaluation of ∆K were performed with STRUCTURE HARVESTER [100]. We then used CLUMPAK web server [101] to find the major and minor best alignment of the results across the range of K values by averaging the probabilities of each K cluster. Graphs were visualized in CLUMPAK [101] and the major mode solution was selected.

For further assessment of population differentiation, the multivariate DAPC method was applied using the *adegenet* 2.1.1 package [102] implemented in R 2.14 [103]. DAPC defines clusters using the algorithm *k-means* on transformed data with Principal Component Analysis (PCA), which is then run sequentially with increasing values of *k*. The resulting clustering solutions are compared using Bayesian Information Criterion (BIC), with the optimal cluster solution corresponding to the lowest value of BIC. Before performing the analysis, the optimal number of PCs to be retained was explored by a cross-validation method as implemented in the same package.

Population differentiation between pairwise sampling sites was estimated with the *F*_ST_ statistic using an infinite allele model (IAM) in the software Arlequin 3.5 [104]. Significance of *F*_ST_ values was analysed with 20,000 permutations and corrected using the B-Y [105] False Discovery Rate (FDR) approach as described in [106]. In addition, the frequency of null alleles was estimated with Microchecker 2.2.3 [107]. Only the microsatellite 17PETRO contained null alleles, and we corrected allele frequencies and *F*_ST_ values after it, using the ENA method [108] described in FreeNA [109]. Global *F*_ST_ and average *F*_ST_ per population following [110] with UPGMA clustering of populations were obtained using the R packages *adegenet* 2.1.1 [102] and *hierfstat* v0.04–22 [111].

To determine whether genetic differentiation was driven by geographical distance creating a pattern of IBD, linearized pairwise *F*_ST_ estimates (*F*_ST_ /1- *F*_ST_) were correlated against log-transformed geographical distances between samples [112] using a Mantel test with all the sites together and stratified Mantel tests using the clusters separated by oceanographic barriers obtained below in GENODIVE version 2.0b23 [95]. Geographical distances were estimated as the minimum linear distance between pairs of locations by sea. Furthermore, to localize the occurrence of genetic breaks in the population structure of *P. ficiformis* (i.e., oceanographic fronts), pairwise *F*_ST_ values and coordinates for sampling sites were implemented in the software BARRIER v2.2 [113]. BARRIER links the matrix of geographical coordinates with their corresponding distance matrix (*F*_ST_), and applies the Monmonier’s maximum distance algorithm to identify ‘barriers’ to gene flow among sites, namely the zones where differences between pairs of sites are the largest.

Finally, an Analysis of Molecular Variance (AMOVA) was performed to determine the hierarchical distribution of genetic variation in GENODIVE version 2.0b23 [95]. To reveal the source of variation for the genetic differentiation, we a priori defined several groupings: 1) two groups: Atlantic (SMI, FLO, CAN, MAD) vs. Mediterranean (CART, CAR, BLA, FEL, ULL, ESC, MRS, NIZ, LIG, NAP, SLO, SCRO, JECRO, CRE, ISR) populations; 2) three groups: Atlantic (SMI, FLO, CAN, MAD), Western Mediterranean (CART, CAR, BLA, FEL, ULL, ESC, MRS, NIZ, LIG, NAP), and Eastern Mediterranean (SLO, SCRO, JECRO, CRE, ISR) populations; and 3) four groups: Azores (SMI, FLO), Madeira and Canary Islands (CAN, MAD), Western Mediterranean (CART, CAR, BLA, FEL, ULL, ESC, MRS, NIZ, LIG, NAP), and Eastern Mediterranean (SLO, SCRO, JECRO, CRE, ISR) populations. The significance of the AMOVAs was calculated with 10,000 permutations of the original data.

### Demographic events and migration patterns in *Petrosia ficiformis*

Given the documented episodes of mass mortalities for *P. ficiformis* [3, 10] in the Mediterranean Sea, we tested for recent effective population size reductions (bottlenecks) based on allele data frequencies using the software BOTTLENECK [114]. The software operates under the assumption that “populations that have gone through a recent reduction of their effective population size show a reduction of the allelic diversity and heterozygosity, even though the number of alleles are reduced faster than the heterozygosity” [114]. We used the “Wilcoxon sign-rank test” [115] which can be used when more than 5 (but less than 20) loci are included. We performed the analyses with two models of mutation: two phase model (TPM) and the stepwise mutation model (SMM), using default values (a proportion of SMM in the TPM = 0.000 and a variance of the geometric distribution for TPM = 0.36), since they are the recommended models for microsatellites.

In addition, a population assignment analysis was performed calculating the likelihood ratio thresholds for all 19 populations based on the Monte Carlo test with an alpha of 0.002 and 1000 replicated datasets using GENODIVE version 2.0b23 [95]. This method assigns or excludes reference populations as possible origins of individuals on the basis of multilocus genotypes. Furthermore, the detection of last generation migrants was performed based on the calculations of the likelihood of an individual belonging to a given population, which was then done replacing the zero frequencies by a random 0.005 frequency in 4000 permutations using GENODIVE. Migration estimates among areas were obtained with diveRsity package (https://diversityinlife.weebly.com/) in R [103], which uses the method described in [116] to plot the relative migration levels between population samples from microsatellite allele frequency data. We only considered relative migration values over 0.1 obtained using the Gst statistic in a bootstrapped analysis (100 replicates). Since the method is still in experimental stages, results should be interpreted with caution. The sampling sites were pooled into the following groups: AZO (SMI, FLO), MAC (MAD, CAN), WES (CART, CAR, BLA, FEL, ULL, ESC, MRS, NIZ, LIG, NAP), ADR (SLO, SCRO, JECRO), EAS (CRE, ISR). To assess long-term migration rates among populations, a coalescent approach using Bayesian implementations in MIGRATE-n was used to obtain both migration rate and theta (θ) [117]: we selected the Brownian model, more appropriate due to the high variability of the microsatellite loci, and then ran 4 replicated chains with an increment of 1500 and 15,000 recorded steps with a 10% burn-in after an initial test run to obtain the correct set of priors for the migration and theta parameters (total visited parameters 1500,000). Convergence was assessed using the effective sample size threshold of 200 and the when the Gelman’s convergence criterion was close to 1. Migration rates were only taken into account when their 95% CIs did not overlap zero.

## Additional files

Additional file 1: Descriptors of genetic diversity for all 19 locations of *Petrosia ficiformis*. Abbreviations: Ns, number of genotyped individuals; A, number of alleles; Ae, Number of effective alleles; rA, number of alleles after rarefaction; Ho, observed heterozygosity; He, expected heterozygosity; F_IS_, inbreeding coefficient; HWE, deviation from Hardy-Weinberg equilibrium: ns, not significant; * = *p* < 0.05; ** = *p* < 0.01; *** = *p* < 0.001. Population codes as in Table 1. (XLSX 58 kb)

Additional file 2: Allele frequencies per locus and population for the entire study. (PDF 1776 kb)

Additional file 3: A. Graph depicting delta K and likelihood of K obtained from STRUCTURE. B. Individual genotype assignment to clusters (K) as inferred by STRUCTURE for all studied sites with K = 10. C. Number of clusters obtained by *adegenet* for *Petrosia ficiformis*. Using Bayesian Information Criterion, the optimal clusters correspond to the lowest values, here shown in red circles. D. Individual assignment to each of the 10 clusters inferred using BIC. (PDF 331 kb)

Additional file 4: *F*_ST_ comparisons between 19 populations of *Petrosia ficiformis*. Bold numbers reflect significant *p*-values after correction (*p* < 0.008). (XLSX 54 kb)

Additional file 5: Migration rates (median and 95% CIs) obtained with MIGRATE-N for *Petrosia ficiformis.* Values with no overlapping CIs are shown in bold and green shading. (XLSX 19 kb)

## Abbreviations

AMOVA: Analysis of Molecular Variance
BIC: Bayesian Inference Criterion
BP: Before Present
CI: Confidence Intervals
FDR: False Discovery Rate
HWE: Hardy-Weinberg Equilibrium
IAM: Infinite allele model
IBD: Isolation By Distance
LGM: Last Glacial Maxima
MPA: Marine Protected Area
Myr: Million Years
PCA: Principal Component Analysis
SMM: Stepwise Mutation Model
TPM: Two-Phase Model
