# Supplementary figures and images for: Genetic diversity, connectivity and gene flow along the distribution of the emblematic Atlanto-Mediterranean sponge Petrosia ficiformis (Haplosclerida, Demospongiae)
Source: BMC Evol Biol. 2019 Jan 16;19:24. doi: 10.1186/s12862-018-1343-6 (PMC6335727; doi:10.1186/s12862-018-1343-6)

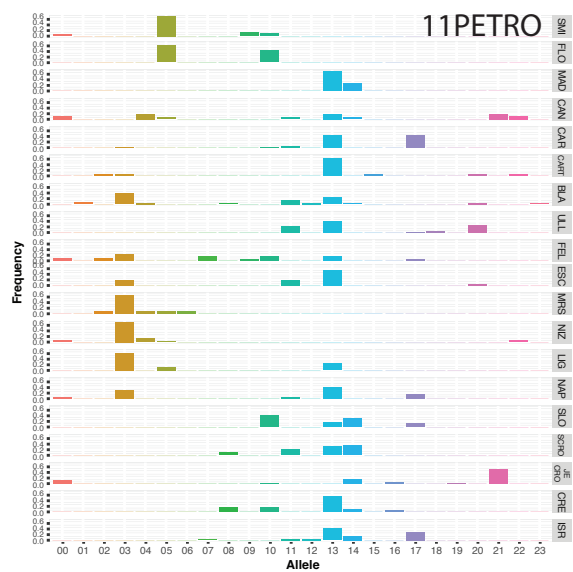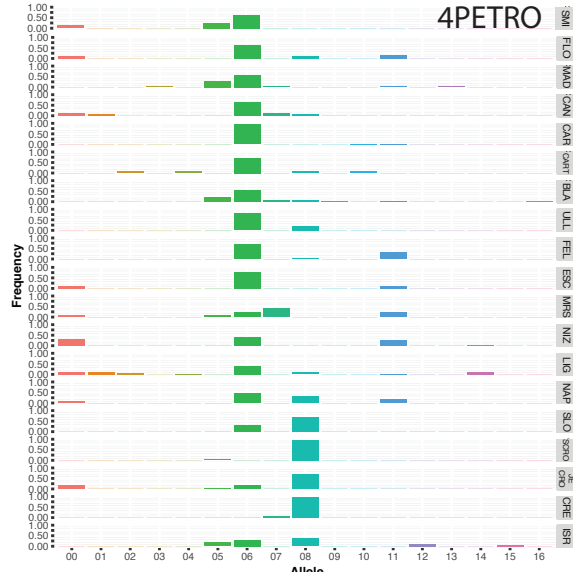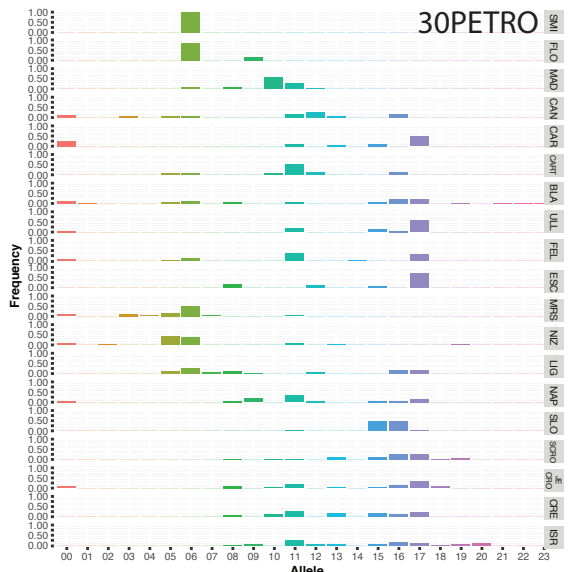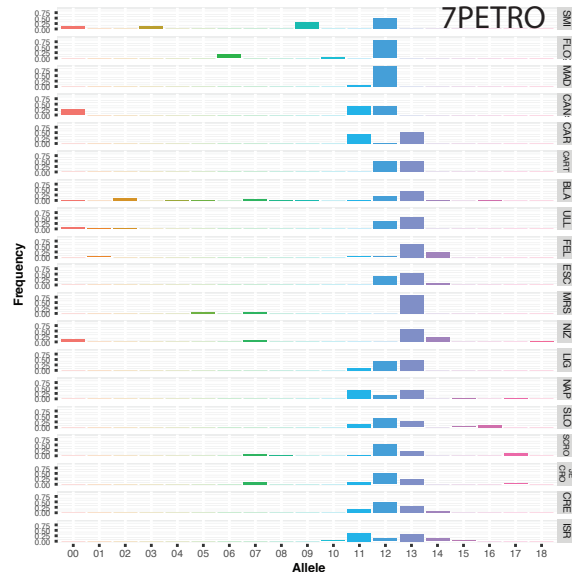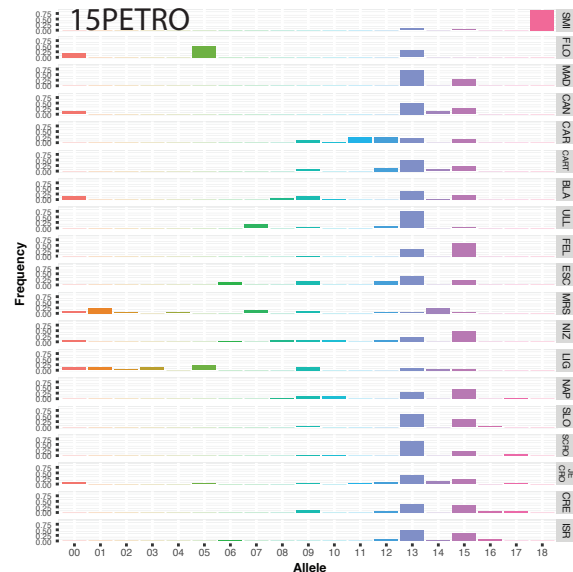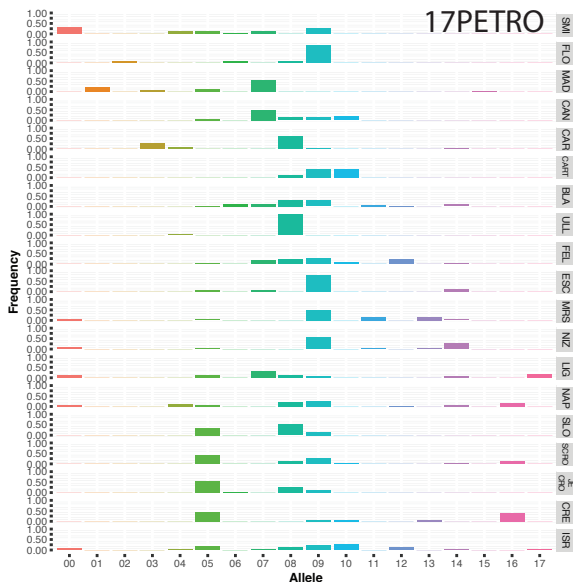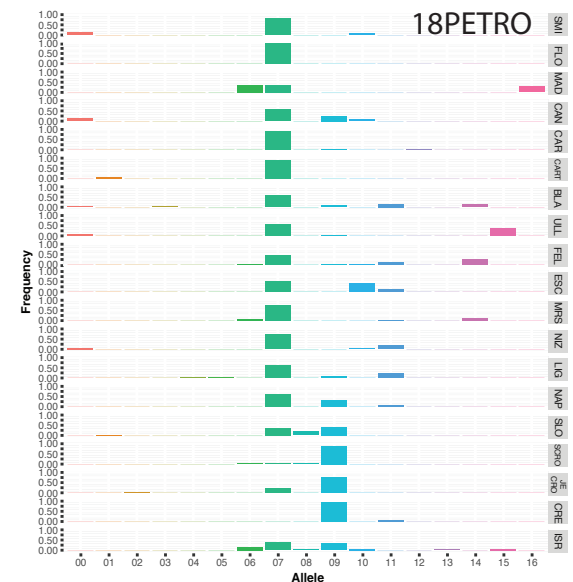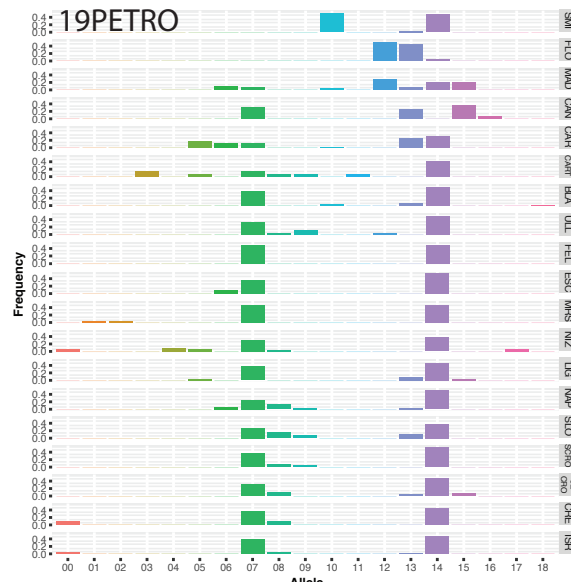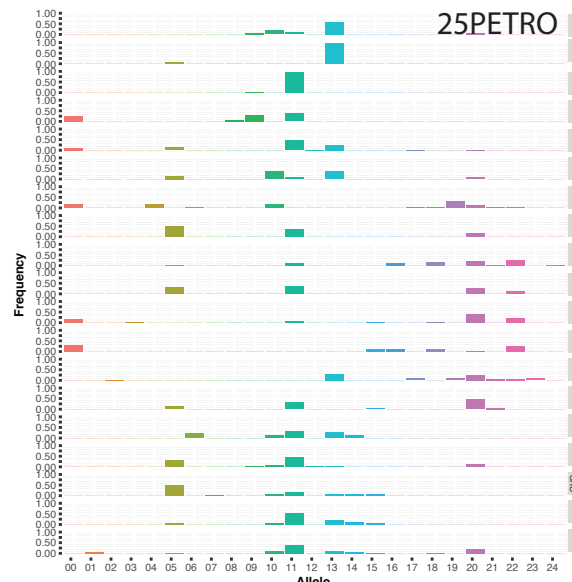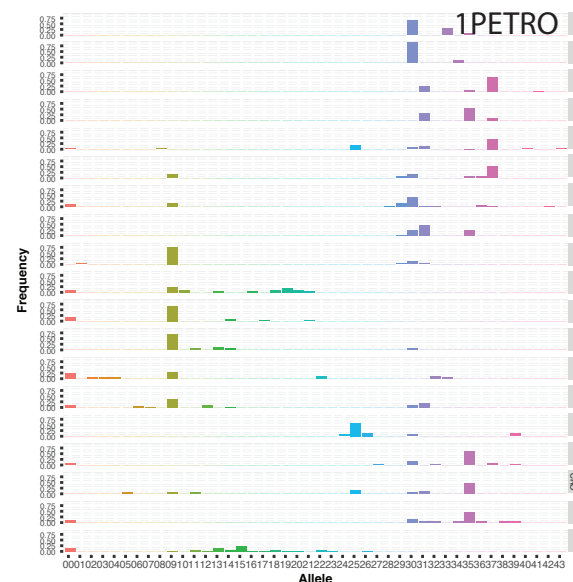

Supplement: Supplementary file 2 — Allele frequencies per locus and population for the entire study. (PDF 1776 kb) [file 12862_2018_1343_MOESM2_ESM.pdf]

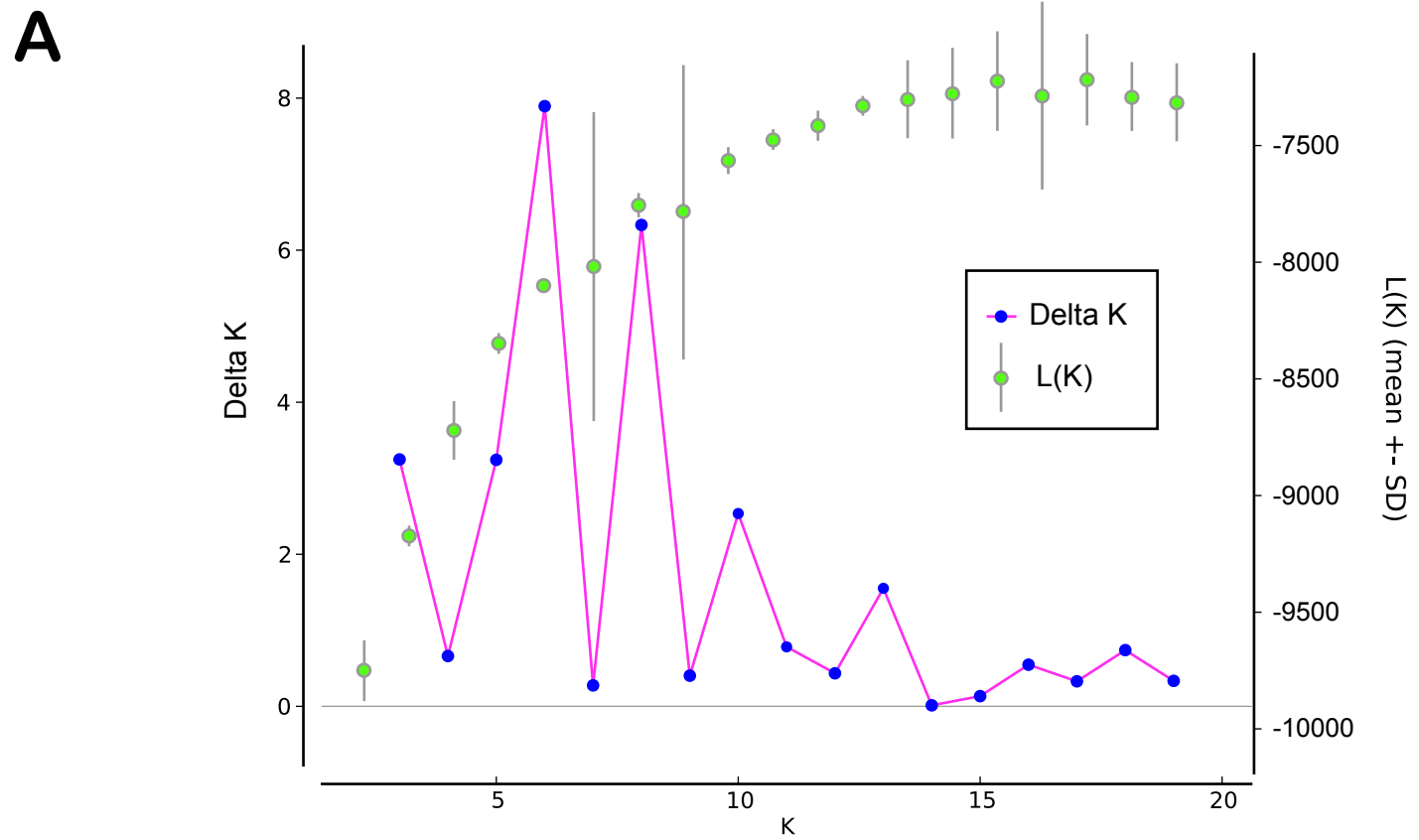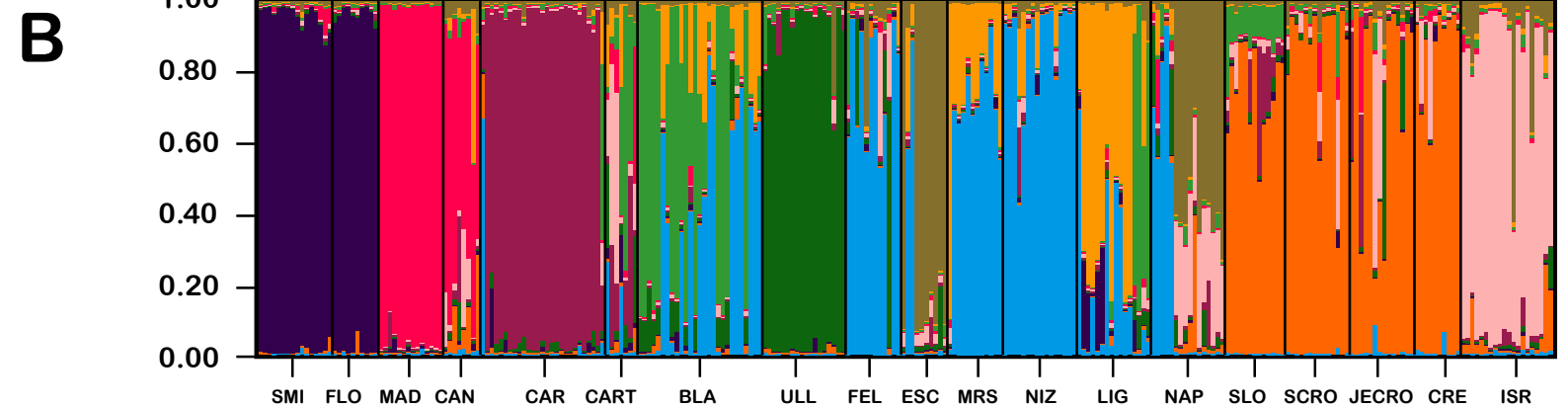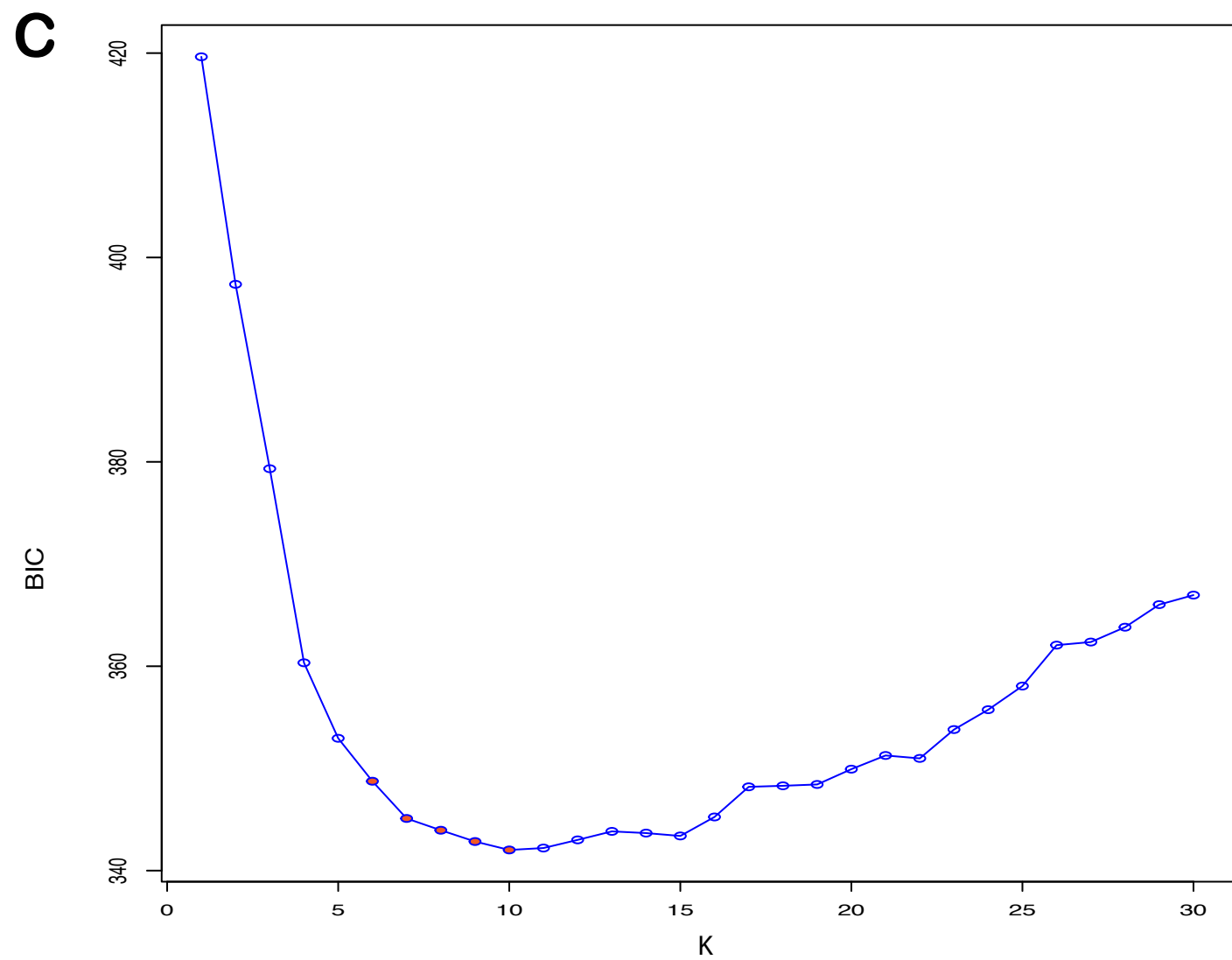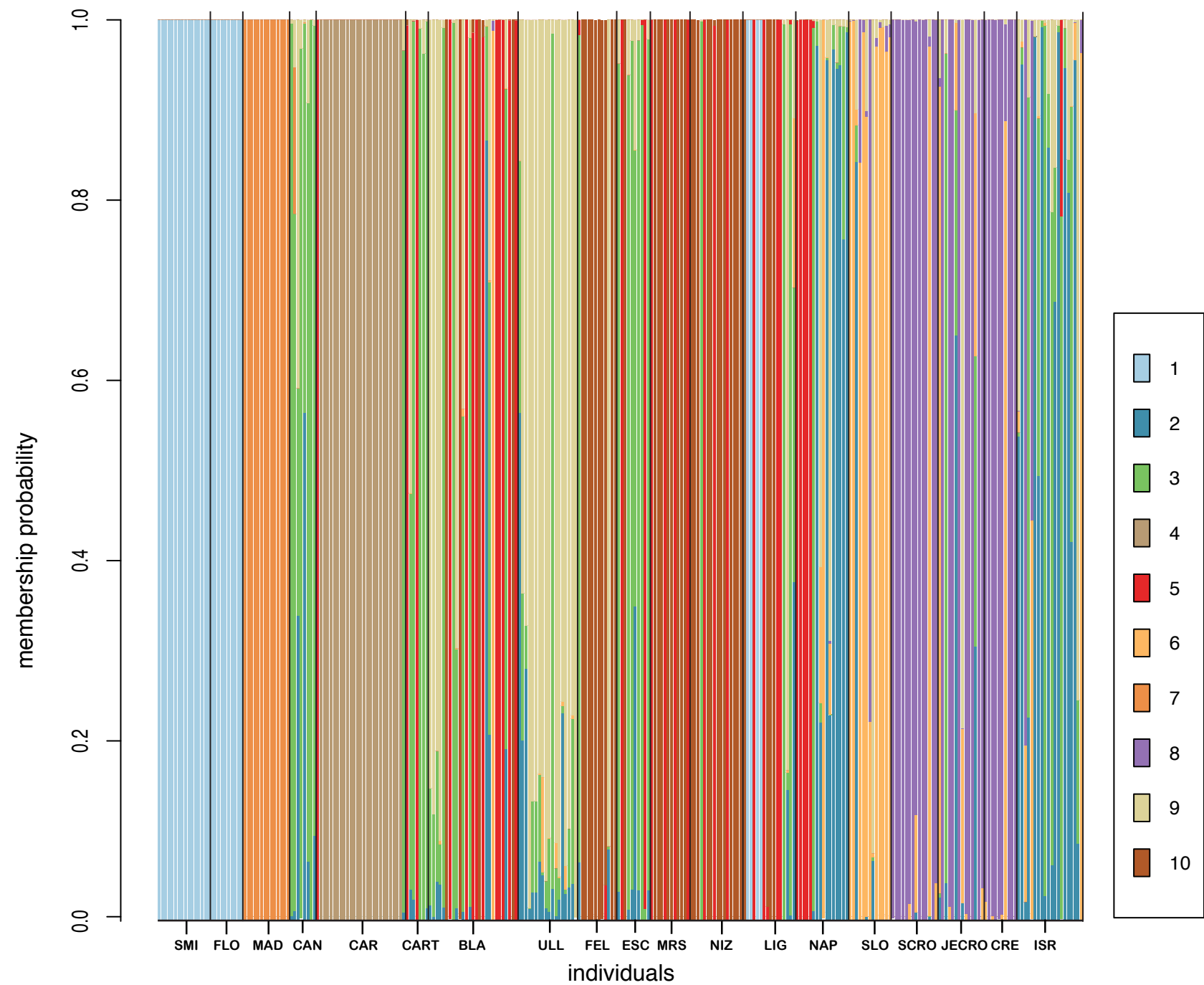

Supplement: Supplementary file 3 — A. Graph depicting delta K and likelihood of K obtained from STRUCTURE. B. Individual genotype assignment to clusters (K) as inferred by STRUCTURE for all studied sites with K = 10. C. Number of clusters obtained by adegenet for Petrosia ficiformis. Using Bayesian Information Criterion, the optimal clusters correspond to the lowest values, here shown in red circles. D. Individual assignment to each of the 10 clusters inferred using BIC. (PDF 331 kb) [file 12862_2018_1343_MOESM3_ESM.pdf]
